# Maternal Experiences with Discussing Complementary Feeding in Primary Care

**DOI:** 10.3390/ijerph191912061

**Published:** 2022-09-23

**Authors:** Kelly Lynn Bouchard, Diana S. Grigsby-Toussaint, Katelyn Fox, Sarah Amin, Maya Vadiveloo, Mary L. Greaney, Alison Tovar

**Affiliations:** 1 Department of Nutrition and Food Sciences, College of Health Sciences, University of Rhode Island, Kingston, RI 02881, USA; 2 Department of Behavioral and Social Sciences, Center for Health Promotion and Health Equity, Brown University School of Public Health, Providence, RI 02912, USA; 3 Department of Health Studies, College of Health Sciences, University of Rhode Island, Kingston, RI 02881, USA

**Keywords:** complementary feeding, infant feeding, primary care, qualitative

## Abstract

Complementary feeding practices promote healthy eating habits and food preferences later in life. Little is known about how US pediatricians communicate infant feeding practices to caregivers or how caregivers respond to this information. The purpose of this study is to explore mothers’ experiences and perceptions of the complementary feeding recommendations they receive in primary care settings. English- and Spanish-speaking mothers of infants were recruited from Special Supplemental Nutrition for Women, Infants, and Children offices in Rhode Island, US, and snowball sampling. Semi-structured telephone interviews were conducted to investigate mothers’ discussions with pediatricians about complementary feeding and their overall impressions of wellness visits. Thematic analysis was informed by the Fundamentals of Care theoretical framework. The mean age of the sample (*n* = 13) was 30.5 years and 62% self-identified as Latina. Four themes emerged from the analysis: (1) wellness visits are mostly positive experiences, (2) not all infant feeding recommendations are easy to follow, (3) alternative sources of infant feeding recommendations can be just as helpful, and (4) there is room for improvement at wellness visits. Improving the content, delivery, and cultural relevance of infant feeding recommendations in primary care settings with more specific and tailored information may promote adherence to evidence-based practices.

## 1. Introduction

High quality nutrition is needed to support rapid physiological and neurological development throughout childhood [1]. Unfortunately, overall diet quality among youth in the US is suboptimal [2,3,4,5]. Given that eating habits in the first 1000 days of life are predictive of health status in later years [6,7], interventions that promote infant diet quality are needed.

The complementary feeding period, the transitional phase between exclusive breastmilk or formula consumption to solid foods [8], is one of the earliest windows for modifying chronic disease risk. When, what, and how solid foods are introduced influence food preferences and body composition into adolescence [9,10,11]. Timely interventions that target complementary feeding practices may facilitate healthful eating habits later in life.

Healthcare providers are uniquely positioned to educate caregivers on childcare [12]. Yet, little is known about US pediatricians’ discussions with caregivers about infant feeding. Limited research suggests that there is low adherence to some of the American Academy of Pediatrics’ infant feeding guidelines [13,14]. Similarly, few studies have explored US caregivers’ attitudes toward the complementary feeding recommendations they receive from pediatricians. There is a need to better understand caregivers’ perspectives on these discussions to inform infant feeding interventions in primary care settings.

Theoretical frameworks are useful for establishing a consistent lens of interpretation, but few have been applied in pediatric primary care. The Fundamentals of Care (FOC) is a theoretical framework that was developed to define patient-centered nursing in acute care settings [15]. The FOC characterizes care as having three interconnected dimensions: the nurse-patient relationship; the integration of physical, psychosocial, and relational care; and the context in which care is delivered. The dynamic components of this framework are intended to guide nurses in optimizing care plans for their patients’ unique needs. There is potential to utilize the FOC to better understand pediatricians’ performance in communicating about infant feeding.

Few studies have explored what recommendations caregivers receive from pediatricians, how they are delivered, and their perceptions of these recommendations. Therefore, the aim of this study was to explore mothers’ experiences and their perceptions of the complementary feeding recommendations they receive in the primary care setting.

## 2. Materials and Methods

### 2.1. Recruitment

Recruitment flyers in English and Spanish were sent to three offices of the Special Supplemental Program for Women, Infants, and Children (WIC) in Rhode Island, US from September 2020 to February 2021. If mothers expressed interest in participating to WIC staff, their contact information was sent to the research team with their permission. Interested mothers also were identified via snowball sampling [16]. Eligibility screening was conducted via telephone. Individuals were eligible if they were at least 18 years old, had an infant between 0–12 months of age that did not have a feeding disorder, and were able to speak English or Spanish. Participants were then scheduled for telephone interviews within a month of the screening date. The anticipated sample size was 10 to 20 participants based on prior qualitative studies with mothers of infants [17,18]. Recruitment ended when data saturation was determined through an empirical evaluation of the depth of code exploration and understanding [19].

Contact information was obtained from 19 mothers who expressed interest in participating, but 6 did not respond to follow-up. A total of 13 mothers, 7 recruited from WIC outreach and 6 recruited from snowball sampling, were interviewed.

### 2.2. Data Collection

An interview guide (Table 1) was created by KLB, DSGB, and AT based on previous infant feeding literature and key concepts from the FOC to gain a better understanding of how pediatricians’ conduct affects mothers’ experiences in primary care settings. It was revised based on pilot test feedback from an eligible non-participant, then translated into Spanish.

Thirteen semi-structured interviews were conducted via telephone in the participant’s preferred language (English or Spanish) between October 2020 and February 2021. Interviews were audio-recorded with Call Recorder iCall (Appitate LLC, Sunny Isles Beach, FL, USA). They lasted from 25 to 67 min, with an average of 40 min. Participants were asked to complete a brief online survey following their interview to collect sociodemographic information, including age, race/ethnicity, education, household income, and federal nutrition assistance enrollment. Infant sex, age, and age of introduction to solid food was also reported. Participants received a $50 gift card as remuneration via email after completing the survey.

### 2.3. Data Analysis

A thematic analysis was conducted following the seven-phase process described by Lester et al. [20]. The interview recordings were deidentified and transcribed by two research assistants. An initial set of codes was derived from the research question and FOC framework prior to analysis for deductive coding. An inductive approach guided by Grounded Theory also was utilized [21], with the codebook being reviewed and updated throughout the analysis period to reflect newly identified recurring concepts. All interviews were independently coded by KLB and a research assistant with MAXQDA (Verbi Software, Berlin, Germany) software. Transcripts underwent multiple readthroughs to promote familiarization with the data. Applied codes were organized into preliminary themes by KLB that evolved with further analysis. Weekly meetings between KLB and the research assistant were held to review coding discrepancies, establish intercoder agreement, and discuss preliminary impressions. Codes were then incorporated into finalized themes and subthemes that summarize their interrelations within the study’s context. Post-interview surveys were created and distributed through REDCap 8.10.1 (Vanderbilt University, Nashville, TN, USA). Demographic information was processed in SAS 9.4 (SAS Institute, Cary, NC, USA).

### 2.4. Ethical Considerations

All aspects of data collection and storage were disclosed to participants. Verbal consent to participate was obtained before interviews commenced. The University of Rhode Island’s Institutional Review Board approved of all study procedures.

## 3. Results

Participants had a mean age of 30.5 years (SD ± 7.2), and 61.5% self-identified as Hispanic (Table 2). Most mothers reported graduating from high school or earning a graduate degree. More than three-quarters were recipients of WIC benefits at the time of the interview. More than a third of the infants had been introduced to solid foods at 4 months of age.

Four themes emerged from the analysis: (1) Wellness visits are mostly positive experiences, (2) Not all infant feeding recommendations are easy to follow, (3) Alternative sources of infant feeding recommendations can be just as helpful, and (4) There is room for improvement at wellness visits. The following section contextualizes these themes within the FOC, then presents subthemes and supporting quotes (Table 3).

### 3.1. Wellness Visits Are Mostly Positive Experiences

Mothers largely held positive opinions about their infant’s wellness visits and the feeding recommendations they received during visits. Some perceived the information to be useful, but specific qualities of relationships with their pediatricians underlie much of the mothers’ satisfaction.

#### 3.1.1. Some Recommendations for Feeding Infants Are Helpful

Mothers who were advised by their pediatrician on the appropriate timing of complementary feeding had neutral responses to these recommendations. However, discussions about what types of foods to introduce were regarded with greater enthusiasm. Infant cereals, puréed fruits and vegetables, avocados, and commercial infant foods were all suggested as items to offer first. This information was especially valuable for first-time mothers who were unfamiliar with their feeding options:

“*I hadn’t even thought of the baby cereal and when she told me I was excited*…” (Participant 7, English)

Others reported that pediatricians supported their autonomy to choose from a variety of foods:

“*I think what they said was a lot of parents choose to start with the cereals, but they said… if we chose to start with vegetable purée… that would be okay, too*.” (Participant 8, English)

Several mothers mentioned that guidance on treating digestive issues was helpful:

“*… [the pediatrician] said that my child was constipated and to give him prune juice with apples, I believe. That helped my child a lot.*” (Participant 2, Spanish)

#### 3.1.2. The Pediatrician Is Knowledgeable about Infant Wellness

Many mothers stated that they trusted what pediatricians told them about caregiving practices, including information on nutrition and feeding during infancy. They elaborated that their trust stemmed from a belief that pediatricians know more about child health than them:

“*…they are professional and you have to follow what professional people advise you because it is good information… It is a pediatrician, and that is someone who has an interest in the well-being of your child.*” (Participant 2, Spanish)

**Table 3:** Themes, subthemes, and supporting quotes from interviews with mothers of infants.

Theme	Subtheme	Supporting Quotes
Wellness visits are mostly positive experiences	Some recommendations for feeding infants are helpful	“It’s actually like they do take their time, like if you’re there for an infant, they do take their time and explain things to you.” (Participant 3, English) “Yes, she always told me what I could feed my baby, what I would give him… [the pediatrician] would talk about all that.” (Participant 1, Spanish) “But on the other hand, the pediatrician has accurate questions, right? Like what you should really know in terms of nutrition and all of that… so, like, I felt more empathy, right?” (Participant 3, Spanish)
	The pediatrician is knowledgeable about infant wellness	“But, you know, I’m believing the doctor knows a little bit more than me, this is what he went to school for.” (Participant 4, English) “Usually, like, we consider doctors to be very smart people…” (Participant 5, English) “What I mean is [feeding recommendations] that would make me feel safer and that came from the pediatrician.” (Participant 3, Spanish)
	Having a good relationship with the pediatrician put mothers at ease	“I feel that it helps that… this pediatrician, it was my pediatrician before it became my son’s pediatrician. I don’t know, it just makes me feel better.” (Participant 5, English) “I mean, my doctor is already pretty personal with me… But I know that other people aren’t so lucky.” (Participant 6, English) “I have a good relationship with her, my daughter’s doctor. Um… yeah, so she’s super helpful.” (Participant 7, English)
Not all infant feeding recommendations are easy to follow	Pediatricians’ infant feeding recommendations can be vague	“…I’m taking WIC but, you know, that’s not the case for every person. So if I wouldn’t have received that… [the pediatrician’s recommendations] would have been very broad. You know, like, not specific enough, at least for someone who is just starting with their baby.” (Participant 1, English) “The doctor didn’t go into specifics or give a brochure to, you know, focus on these kind of food groups for now.” (Participant 2, English) “I feel like they generalize too much.” (Participant 3, Spanish)
Not all infant feeding recommendations are easy to follow	Every child has different feeding needs	“Every child is different, you know, and… some wanna eat before, you know, some eat before others eat…” (Participant 4, English) “A baby could be ready for solid foods at four months or five months or five and a half. You don’t have to wait until six months to introduce solid foods.” (Participant 5, English) “Everybody’s different, so one person’s advice might not be for your kid, only for theirs.” (Participant 10, English)
	Infant feeding recommendations do not account for cultural differences	“…we’re Hispanic, so the baby food that they sell on the market, it’s not going to be the same as to what we put in the house.” (Participant 2, English) “Like, in the Hispanic culture, a lot of people start feeding them cereal when babies seem not to get full on milk? …so we tried that for, like, maybe three weeks with one of the babies… But the doctor noticed. The doctor’s like, ‘you can feed them food but don’t- it’s not time for [cereal].” (Participant 9, English) “[Immigrants] don’t have much support. We don’t have people close to us, and all that stuff which makes it stronger and frustrating for us.” (Participant 3, Spanish)
Alternative sources of infant feeding recommendations are sometimes preferable	The pediatrician is not always the nutrition expert	“… I talk more with WIC when it comes to, you know, the children’s feedings than the pediatrician.” (Participant 4, English) “So it’s annoying, so I tend to call the WIC office and they send the handouts… So I’ve kinda been asking them more than my doctor.” (Participant 9, English) “Sometimes it’s better to hear it from another mom than a doctor that doesn’t even have kids and they’re going off of, like, textbook.” (Participant 10, English)
	Family members are convenient sources of infant feeding recommendations	“But my mom has been super huge help in that sense… Like, she knows what she’s doing. So I, you know, would watch her and just learn from her in that sense.” (Participant 1, English) “Yes, so, because I had experience from my mother-in-law.” (Participant 1, Spanish) “As always, my mother-in-law- my mother-in-law, since I asked, gives me advice.” (Participant 2, Spanish)
Alternative sources of infant feeding recommendations are sometimes preferable	Personal research fills in gaps in infant feeding knowledge	“Like, obviously what we learn is also through the internet. Like reading and, you know, researching and I guess that influenced us to think that after six months that was, you know, something we should do.” (Participant 1, English) “Like, everything is, y’know, you just search it and there’s a million answers for you out there.” (Participant 5, English) “…I had googled what foods you cannot give a baby and you can’t give honey to a baby that’s less than one year old. So I feel like… how would they know that? Unless, like, the doctor tells them. So, y’know, do your research. If the doctor isn’t telling, if you have questions, but, y’know.” (Participant 7, English)
There is room for improvement at wellness visits	There are barriers to following pediatricians’ recommendations	“So I didn’t necessarily- obviously, I didn’t even go by what the handout said, because the handout did not say anything about baby-led weaning in it.” (Participant 8, English) “I’ll take a little notebook or I’ll take note on my cellphone. But sometimes I do forget, y’know, was [the pediatrician] talking about this baby, or what was it that he said?” (Participant 9, English) “So in this case, in this country it is very hard to take [infants] wherever you go because you also have to work. You have to leave them.” (Participant 1, Spanish)
	Mothers have varying opinions on how wellness visits could be improved	“I don’t know, I guess if there’s some form of a photocopy or a paper, it’d be easier for [the pediatrician] just to hand it over and write down whatever might be specific to my child.” (Participant 1, English) “Even, like, a website. It could be a website. Hey, y’know, here’s this website that you could refer to this or, y’know, there’s this interactive platform where you can ask questions and we’ll answer or look at these pamphlets, or something like that.” (Participant 9, English) “I feel like a couple years ago used to be a lot more intimate, where it’s not now. Like, they just kind of rush you off to the next patient, which I understand they’re busy… So, I do get it, but I just kind of wish it was the way it used to be.” (Participant 10, English)

#### 3.1.3. Having a Good Relationship with the Pediatrician Put Mothers at Ease

Having rapport with their child’s pediatrician also influenced mothers’ wellness visit experiences. Many mothers were familiar with the pediatrician through family members who attended the same primary care facility or from when they were patients themselves during their childhoods. Mothers described how having a history with the pediatrician was reassuring:

“*I like my doctor because it’s my kids’ doctor and my doctor, all the once so, I’ve had her for a long time.*” (Participant 10, English)

One mother who had to change pediatricians explained how her current provider relationship was not as satisfying:

“*Well, for my first son, my three-year-old, I had my doctor, another doctor, and he recently retired, like, maybe two years into my son’s life, and then this new doctor had took over… So, I don’t know if you know, I miss the old one. He’s nothing like the older one.*” (Participant 4, English)

Mothers shared that relationships with their pediatricians were established and maintained through active provider support. They spoke of providers affirming their decisions:

“*It just seems like they support the decisions that we’ve been making, which is always good to have from your pediatrician.*” (Participant 8, English)

Asking about their well-being, answering questions, providing infant formula samples, and writing medical notes to promote breastfeeding in the workplace as examples of provider support, which were appreciated.

### 3.2. Not All Infant Feeding Recommendations Are Easy to Follow

Despite stating that their pediatrician’s feeding recommendations were helpful, most mothers also shared that there was some information that they were dissatisfied with or did not follow. They elaborated that pediatricians failed to meet their standards of clarity, specificity, and cultural competence.

#### 3.2.1. Pediatricians’ Infant Feeding Recommendations Can Be Vague

Most mothers felt that their discussions about infant feeding in primary care were not clear enough or could be vague or generic:

“*But what I feel is… sometimes we tend to generalize a whole process where [the pediatrician] could be… like [recommendations] could be different but they are the same…*” (Participant 1, Spanish)

Mothers relied on their intuition to determine how to feed their infants when the recommendations they had received were ambiguous. This maternal instinct was sometimes used to gauge when their infant was ready for solid foods. One mother shared:

“*But he recently turned six months old, and my intuition told me that it was time to start feeding him small things… like to start something different.*” (Participant 3, Spanish)

Some mothers spoke of pediatricians supporting their decisions to initiate or delay complementary feeding:

“*So at the four month visit, they said we could start [introducing solid food] at that point. And I said I wanted to wait and they were fine with that. So they didn’t necessarily recommend that at that point, I guess.*” (Participant 8, English)

Many mothers also reported choosing what foods to feed their infants without a pediatrician’s input.

#### 3.2.2. Every Child Has Different Feeding Needs

Some mothers felt that providers’ feeding recommendations did not accommodate their infants’ unique needs and behaviors. Specifically, the signs of readiness for solid foods, hunger cues, and food preferences that mothers reported observing in their children sometimes conflicted with the information pediatricians offered about when and how to feed their infants:

“*Like, I definitely listen to the doctor and, okay, this is what the doctor told me, y’know, let’s work toward that. But it’s not, like, a given because… clearly not everyone’s the same and so on.*” (Participant 9, English)

These discrepancies convinced some mothers to rely on infant feeding methods that their pediatrician had not mentioned:

“*…as long as the baby is happy, healthy, and growing as they should be, I think that there are other healthy methods than necessarily what the doctors might recommend.*” (Participant 8, English)

#### 3.2.3. Infant Feeding Recommendations Do Not Account for Cultural Differences

Mothers from ethnically diverse backgrounds spoke of how culturally influenced practices impact how they feed their infants. For instance, one mother described her parents’ approach to infant feeding:

“*They were actually saying you can do this! You can do anything! Feed her some of this. Feed her some of that. They’re also Arabic so, y’know, their culture. How they grew up.*” (Participant 7, English)

Beyond acknowledging familial influences on attitudes toward feeding, these mothers attempted to incorporate culturally relevant foods into their infant’s diet. Yet, some explained that it was difficult to balance their food preferences with infant feeding recommendations in the US:

“*But it is hard to adapt to the idea of… don’t give tomatoes but give apples or don’t give apples but give carrots… Do you know what I mean?*” (Participant 2, Spanish)

Some mothers noted that these cultural differences affected how they responded to caregiving information from primary care settings.

### 3.3. Alternative Sources of Infant Feeding Recommendations Are Sometimes Preferable

All mothers reported receiving infant feeding recommendations from sources beyond primary care, including relatives, other mothers, WIC, and the internet when they felt that their pediatrician did not offer sufficient feeding guidance.

#### 3.3.1. The Pediatrician Is Not Always the Nutrition Expert

Some mothers sought information from alternative sources because they did not consider their pediatricians to have adequate nutrition knowledge compared to other available resources. This was often because mothers reported feeling that nutrition counseling was not within their pediatrician’s scope of practice:

“*I see, like, my child’s pediatrician more as someone that I could address for some form of a medical concern you- like, some form of a physical reaction or behavioral… behavior that might be concerning, not really as someone I can ask about her nutrition.*” (Participant 1, English)

Mothers spoke of WIC and other caregivers being preferable to consult about complementary feeding due to their perceived expertise in nutrition and experience with feeding, respectively.

#### 3.3.2. Family Members Are Convenient Sources of Infant Feeding Recommendations

Mothers frequently cite family members, especially female relatives, as sources of feeding advice. Some mothers explained that they preferred to ask family members about feeding concerns before contacting a pediatrician:

“*First I would ask my mother before I called the pediatrician ’cause sometimes she would know. She would have a solution (laughs) before it escalated to the pediatrician.*” (Participant 5, English)

They described this information as helpful:

“*[My mother]’s the one that has [been] more helpful to both of us, both my sister and I, like cooking certain foods for both babies.*” (Participant 1, English)

#### 3.3.3. Personal Research Fills in Gaps in Infant Feeding Knowledge

Some mothers described searching for additional information that the pediatrician did not mention. One mother discussed how she learned most information about baby-led weaning from her own research:

“*You know, I wasn’t even necessarily talking to anybody, I was just reading [online] stories, and doing some research online too and that is where I got most of my advice and information.*” (Participant 8, English)

Research also was used to corroborate advice received from multiple sources:

“*I’ll listen to my friend. I’ll listen to the doctor. I’ll listen to even the WIC office ‘cause they get asked about all these feeding questions all the time. I’ll look at where my girls are at. And then I’ll kinda… use a little bit of reasoning. And then my reasoning usually follows up with some research.*” (Participant 9, English)

Mothers additionally mentioned common allergens and inappropriate foods for infants as topics they researched.

### 3.4. There Is Room for Improvement at Wellness Visits

Some mothers commented on broader social and environmental contexts that affect their response to their pediatricians’ recommendations. In addition, mothers were asked to evaluate their experiences in pediatric primary care and consider what changes they would like to see implemented to improve discussions about complementary feeding.

#### 3.4.1. There Are Barriers to Following Pediatricians’ Recommendations

A few mothers mentioned that busy lifestyles or environments interfered with their ability to follow their pediatrician’s recommendations. Some of these distractions were present at wellness visits:

“*So it is a little overwhelming trying to deal with the baby while I’m trying to listen to this information and [the pediatrician] does speak a little bit fast.*” (Participant 1, English)

Returning to work also was described by several mothers as being disruptive to infant feeding.

A lack of culturally sensitive resources also was identified as a barrier by the Spanish-speaking mothers. One mother who immigrated to the US shared her emotional struggles as she transitioned to a new cultural climate:

“*All the fear and sadness because we obviously… the fear we have is very strong, and I feel like mostly the immigrants are going through… or we feel lonelier, to call it something.*” (Participant 3, Spanish)

This mother elaborated that these feelings and a language barrier impacted her ability to follow her pediatrician’s recommendations. The difficulty of navigating another culture was echoed by another mother from Central America:

“*…and the problem is that we have a lot of differences… remember that we are… we are in a country where the culture is very different.*” (Participant 2, Spanish)

Some mothers described COVID-19 prevention measures in primary care facilities, such indoor mask mandates, temperature checks, and revised waiting room policies. None stated that these protocols impaired the quality of their wellness visits.

#### 3.4.2. Mothers Have Varying Opinions on How Wellness Visits Could Be Improved

Mothers presented ideas on how wellness visits could be improved. Many mothers requested that more take-home materials be provided:

“*Not just about, y’know, necessarily feeding, but in general, y’know, any resources would be welcome.*” (Participant 7, English)

They discussed how these materials would be easy to reference if they had difficulty recalling their discussions with the pediatrician. Aside from additional resources, there was no consensus on what modifications to primary care were most desired. Responses varied from prioritizing the caregiver’s concerns, increasing engagement with the healthcare team, and having more discussions about developmental milestones.

## 4. Discussion

The goal of this study was to explore mothers’ experiences and their perceptions of the complementary feeding recommendations they receive in the primary care setting. The FOC guided the organization of their perspectives into four main themes. Overall, mothers expressed positive opinions of wellness visits and their infant’s pediatricians but felt that feeding recommendations they received in this setting were lacking. Moreover, they were comfortable relying on alternative sources of advice in conjunction with their pediatrician when they sought additional information. Mothers had varying opinions on how wellness visits could be improved but generally desired more take-home materials about infant care and feeding.

Mothers largely attributed their satisfaction with their primary care experiences to the relationships they developed with their pediatricians. Patient-provider relationships are at the core of the FOC framework [15]. They serve as the lens through which providers can gain a comprehensive understanding of the patient’s needs. Quality care is then upheld via relational aspects of care delivery as described in the framework’s second dimension. Prior studies have established that mothers seek infant care advice from pediatricians more than they do from other sources [12,22,23]. This trust is influenced by the perception that pediatricians are authorities on child health. For example, one study found that mothers were more responsive to information from pediatricians if they believed that pediatricians were qualified [24]. Beyond establishing trust, the current study found that these relationships are strengthened by familiarity and empathy. Low-income mothers have previously described being less receptive to recommendations from pediatricians if they felt that the interaction was impersonal [25,26]. Pediatricians appear to easily establish trust due to expectations of their knowledge, but rapport can be undermined if they fail to meaningfully engage with caregivers [27]. Utilizing patient-centered communication strategies when delivering infant feeding information may promote caregiver satisfaction and adherence to recommendations [28].

Despite expressing their trust in pediatricians, most mothers reported that some infant feeding recommendations were difficult to follow. According to the FOC framework’s second dimension, the integration of care, providers must simultaneously meet the physical, psychosocial, and relational aspects of a care task [15]. Mothers explained that the advice they received was sometimes unclear, not tailored to their child, or not culturally sensitive, indicating that their psychosocial communication needs were not met. This is consistent with prior studies [29,30], in that mothers have ignored feeding advice that they did not consider to be in their child’s best interest [17,25,31]. Incongruence between complementary feeding recommendations and cultural preferences has also been documented. Mothers’ infant feeding practices are often rooted in their community’s caregiving norms [32,33]. Culturally diverse and immigrant mothers have varied opinions on the importance of evidence-based guidelines [34,35], but they report similar struggles in navigating information from conflicting sources [36,37,38]. It is imperative that pediatricians deliver nuanced and culturally competent recommendations to prevent mothers from making unfavorable decisions.

Mothers reported that they often sought complementary feeding advice from other sources. This is an expected response to unfulfilled care needs because disruptions to care integration place strain on the patient-provider relationship [15]. In the current study, pediatricians’ nutrition information was less valuable to mothers than what could be learned elsewhere. Other studies have reported that mothers favor advice from caregivers with experience over official guidelines [22,39]. Anecdotal evidence is not guaranteed to promote optimal health outcomes, but pediatricians have acknowledged a need to improve their own complementary feeding knowledge [14]. Female relatives and the internet were cited as prominent influences on mothers in the current study, which is consistent with the literature on infant feeding resources [23,40]. Grandmothers can be positive caregiving role models [41], but trusting advice from family members who are unfamiliar with best practices can promote unhealthy child feeding behaviors [42]. Mothers have been more hesitant to accept advice from websites and online forums, though they continue to access these platforms out of convenience [43]. Maternal intuition appears to be a universal metric for making final judgments on the validity of complementary feeding information [44]. However, such “instincts” can lead to poor feeding decisions if there are gaps in caregiving knowledge. This further emphasizes the need for pediatricians to elaborate on the significance of their recommendations to promote adherence without diminishing the value of experiential knowledge.

Several barriers to following pediatrician recommendations were identified. Some of these difficulties stem from within the contextual layer of the FOC framework [15]. Shortcomings in the policies and systems that define the primary care setting can impede mothers’ ability to follow best practice recommendations. For instance, pediatricians may have limited time or nutrition training to address topics of interest in sufficient detail [45,46]. Spanish-speaking mothers also mentioned encountering cultural discordance, a known hindrance to patient satisfaction and adherence to recommendations [47,48]. Offering more educational resources was the most common suggestion to address these obstacles. Pediatricians have also voiced their interest in take-home materials, instructional videos, websites, and similar items [49,50]. Incorporating such resources into primary care would likely be well received by patients and providers alike and should be considered in future interventions.

This study contributes to the limited body of research on US caregivers’ attitudes toward pediatricians’ complementary feeding recommendations. The potential for interpretation bias was minimized by establishing intercoder agreement on all interview transcripts. Several limitations should also be acknowledged. First, not monitoring which primary care facilities the mothers attend means that the experiences shared in this study may reflect a limited number of pediatricians. It is possible that the perspectives of mothers with pediatricians who discuss complementary feeding differently than what was described by this sample were not captured. Similarly, partial reliance on snowball sampling for recruitment increases the risk of sampling bias. The small sample size and exclusive recruitment from one state also limit the generalizability to mothers’ experiences in other regions. Finally, parity was not specified in this study’s inclusion and exclusion criteria even though pediatricians may share different information with primiparous women compared to multiparous women. These limitations are attributed to recruitment challenges that were encountered during the COVID-19 pandemic. However, reaching data saturation indicates that there was sufficient data to perform a complete thematic analysis.

## 5. Conclusions

Despite having positive opinions of pediatric primary care, this sample of mothers did not always consider pediatricians’ infant feeding recommendations to be comprehensive enough to meet their needs. Improving the content, delivery, and cultural relevance of infant feeding recommendations in primary care settings with more specific and tailored information may promote maternal adherence to evidence-based feeding practices. Moreover, the FOC served as a useful framework for describing responses to feeding guidance in primary care. Additional refinement may enhance its use for future studies in this setting. Future research may also explore ways to integrate these new elements into wellness visits with consideration for underlying components of care.

## Figures and Tables

**Table 1:** Complementary feeding interview guide.

1. Could you tell me about your experiences with feeding your infant? a. What was your feeding plan before you had your infant?
2. Could you tell me a little about when you decided to start giving your infant any solid food? a. How did you know when he/she was ready to start eating food? b. Could you describe what some of those first foods were? c. Can you describe the advice/guidance you received about when your infant should start eating solid foods? d. Can you describe the advice/guidance you received about what your infant should be eating as first foods?
3. Can you tell me a little bit about your wellness visits at the pediatrician’s office?
4. What did your pediatrician say about when you should start feeding your infant? a. How was this information given to you? b. Do you remember how old your infant was when you got this information?
5. What did the pediatrician say about what you should feed your infant? a. What foods did they recommend you start with? b. What information did they provide with regards to how much you should be giving your infant? c. What information did they provide with regards to how to feed your child? d. What information did they provide with regards to when to introduce different food textures? e. How long did they spend providing any recommendations/information? f. How was this information given to you? g. Do you remember how old your infant was when you got this information?
6. What are your thoughts on how this information was given to you? a. How did the pediatrician respond to your questions or concerns? b. What concerns about complementary feeding do you have that were not addressed by the pediatrician? c. What resources given to you were the most/least helpful? d. What has influenced your decision to follow/not follow the pediatrician’s recommendations?
7. How has COVID-19 impacted your wellness visit routine? a. How would you compare the quality of visits during the pandemic with other wellness visits you’ve experienced? b. What accommodations have your pediatrician made for visits during the pandemic?
8. If we were to design an intervention, what aspects would be the most important to you as a caregiver? a. What would your ideal wellness visit be like? b. What change to your wellness visits would you most like to see? c. What modes of communication would you prefer to build on your wellness visits?
9. If you were to give other moms advice about infant feeding, what would it be?

**Table 2:** Demographic characteristics of mothers of infants (*n* = 13).

Demographics	Variables	n * or Mean	% or SD
Age		30.5	±7.2
Race	African American or Black	2	15.4
	White	7	53.8
	Other	4	30.8
Ethnicity	Hispanic/Latina	8	61.5
Education	10th–12th grade	1	7.7
	High school diploma or GED	4	30.8
	2-year college degree	1	7.7
	4-year college degree	3	23.1
	Graduate degree	4	30.8
Annual household income	≤$20,000	4	44.4
	$20,001–$30,000	1	11.1
	$30,001–$40,000	1	11.1
	≥$40,001	3	33.3
Nutrition assistance	Receives WIC benefits	10	76.9
	Receives SNAP benefits	3	23.1
Sex of infant	Male	8	61.5
	Female	5	38.5
Age of infant	4 months	1	8.3
	5 months	1	8.3
	6 months	3	25
	7 months	0	0
	8 months	2	16.7
	9 months	3	25
	10 months	0	0
	11 months	2	16.7
Age infant was introduced to solid foods	4 months	5	38.5
	5 months	1	7.7
	6 months	4	30.8
	7 months	1	7.7
	Has not been introduced to solid foods	2	15.4

* Some cells do not add to total n as some participants chose not to answer this question.

## Data Availability

The data analyzed in the current study is available from the corresponding author upon request.
